# Molecular profiling of ex vivo prostate cancer CAF models captures stromal heterogeneity and drug vulnerabilities

**DOI:** 10.1038/s41420-025-02792-3

**Published:** 2025-11-06

**Authors:** Frida Rantanen, Astrid Murumägi, Mariliina Arjama, Katja Välimäki, Elina Multamäki, Tuomas Mirtti, Antti Rannikko, Teijo Pellinen, Daniela Ungureanu, Olli Kallioniemi

**Affiliations:** 1 Disease Networks Unit, Faculty of Biochemistry and Molecular Medicine, University of Oulu, Oulu, Finland; 2 Institute for Molecular Medicine Finland (FIMM), Helsinki Institute of Life Science (HiLIFE), University of Helsinki, Helsinki, Finland; 3 Research Program in Systems Oncology, Faculty of Medicine, University of Helsinki, Helsinki, Finland; 4 HUS Diagnostic Center, Department of Pathology, HUS Helsinki University Hospital, Helsinki, Finland; 5 Finnish Cancer Institute, Helsinki, Finland; 6 Department of Urology, University of Helsinki and Helsinki University Hospital, Helsinki, Finland; 7 Science for Life Laboratory, Department of Oncology & Pathology, Karolinska Institutet, Stockholm, Sweden

**Keywords:** Mechanisms of disease, Tumour heterogeneity

## Abstract

Cancer-associated fibroblasts (CAFs) are central architects of the prostate cancer (PCa) microenvironment, yet their phenotypic diversity and druggable vulnerabilities remain largely uncharted. Here, we present an integrative multi-omics characterization of primary ex vivo CAFs from seven treatment-naïve PCa patients. Using single-cell RNA sequencing (scRNA-seq), we uncover substantial transcriptional heterogeneity among CAFs, with distinct gene expression programs related to extracellular matrix remodeling, inflammation, immune modulation, and metabolic reprogramming. This phenotypic diversity was further supported by variable expression of canonical stromal markers, including FAP, SULF1, VIM, CAV1, and αSMA. Transcription factor network analysis revealed SOX, FOX, and STAT3 family members as key regulators of pro-tumorigenic CAF states. To probe therapeutic vulnerabilities, we performed high-throughput drug sensitivity and resistance testing (DSRT) across 396 oncology compounds. CAFs exhibited broad sensitivity to multikinase inhibitors, with dasatinib, midostaurin, and FGFR inhibitors (AZD4547, erdafitinib) emerging as top stromal-directed candidates. These findings underscore the plasticity of prostate CAFs and reveal actionable vulnerabilities, supporting the development of targeted stromal therapies to disrupt tumor-stroma interactions in PCa.

## Introduction

Prostate cancer (PCa) is among the most frequently diagnosed malignancies and a leading cause of cancer-related mortality in men worldwide [1, 2]. Tumor progression and therapy resistance in PCa are shaped not only by malignant epithelial cells but also by the tumor microenvironment (TME), where cancer-associated fibroblasts (CAFs) play a central role [3–5]. CAFs contribute to multiple tumor-supportive processes, including extracellular matrix (ECM) remodeling, angiogenesis, inflammation, and immunomodulation [6–11].

Strong evidence suggests that CAFs are not a uniform population but consist of phenotypically and functionally distinct subtypes, such as inflammatory CAFs (iCAFs), myofibroblastic CAFs (myCAFs) comprising several subclasses related to their roles in ECM/contractility, adhesion, and cytoskeletal/structural organization, and antigen-presenting CAFs (apCAFs), each with unique paracrine signaling and transcriptional programs [12]. However, a comprehensive understanding of CAF heterogeneity in PCa, including their functional roles and therapeutic vulnerabilities, remains limited—partly due to a lack of well-characterized ex vivo models that preserve patient-specific stromal diversity.

Fibroblast Activation Protein (FAP), a stromal marker overexpressed in many CAFs, including in PCa, has been explored as a prognostic biomarker and therapeutic target [13–15]. Yet, FAP-targeted therapies have shown variable clinical efficacy, likely reflecting underlying CAF plasticity, subtype diversity, and microenvironmental modulation of FAP expression [16–20]. Beyond FAP, signaling pathways such as Galectin-1 (Gal-1), the fibroblast growth factor receptor (FGFR)-Gremlin-1 axis, and glucocorticoid (GR) receptor signaling have emerged as key mediators of tumor–stroma crosstalk, particularly in treatment-resistant disease [21–24].

To address this knowledge gap, we established and functionally characterized a panel of seven primary CAF models derived from treatment-naïve PCa patients. Using an integrative approach, including immunohistochemistry (IHC), single-cell RNA sequencing (scRNA-seq), and high-throughput drug sensitivity and resistance testing (DSRT), we aimed to map the molecular diversity of CAFs, define subtype-specific gene networks, and identify actionable stromal drug targets. This resource provides a foundation for advancing stromal-directed strategies in PCa precision oncology.

## Results

### PCa-derived CAFs exhibit distinct morphology and marker expression in situ and ex vivo

**Fig. 1:**
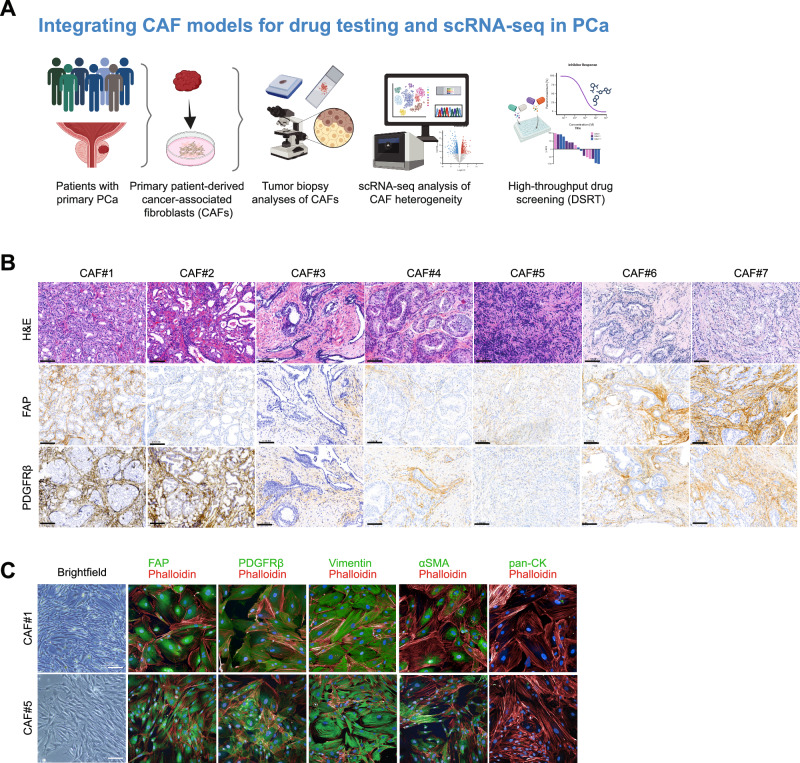
Study overview and phenotypic characterization of PCa–derived CAFs. **A** Schematic representation of the study workflow outlining molecular profiling of ex vivo cancer-associated fibroblasts (CAFs) using immunohistochemistry (IHC), single-cell RNA sequencing (scRNA-seq), and drug sensitivity and resistance testing (DSRT). **B** Representative hematoxylin and eosin (H&E) staining of tumor biopsies from seven PCa patients. Immunohistochemical staining (IHC) for FAP and PDGFRβ is shown below to demonstrate CAF marker expression in tumor tissues. Scale bar indicates 100 μm. **C** Representative multiplexed immunofluorescence (mIF) images of CAF#1 and CAF#5 highlighting the expression of FAP, PDGFRβ, vimentin, αSMA, and pan-cytokeratin (pan-CK), confirming the CAF phenotype. Scale bar indicates 400 μm.

We aimed to establish a platform for the molecular and functional characterization of PCa-derived CAFs (Fig. 1A). Biopsy samples were collected from seven PCa patients undergoing radical prostatectomy. Histopathological analysis of hematoxylin and eosin (H&E)-stained sections revealed varying epithelial-to-stroma ratios across the samples (Fig. 1B, top row, Supplementary Table 1). Immunohistochemical (IHC) staining for CAF markers FAP and PDGFRβ showed prominent expression in the tumor stroma of most samples, particularly CAF#1 and CAF#7, with variable intensity in others (e.g., CAF#3 showing lower FAP/PDGFRβ), confirming the presence and heterogeneity of CAF populations within the primary tumors (Fig. 1B, middle and bottom rows, Supplementary Fig. 1).

Primary CAFs were successfully isolated and cultured from all seven patient-derived tumor tissues. These primary CAFs exhibited the characteristic spindle-shaped morphology with elongated cell bodies and prominent cytoplasmic extensions, consistent with activated fibroblasts (representative images for CAF#1 and CAF#5 shown in Fig. 1C). Immunofluorescence (IF) staining further confirmed CAF identity across all primary cultures. As shown for CAF#1 and CAF#5, cells demonstrated robust expression of FAP, PDGFRβ, and vimentin, along with the myofibroblast marker αSMA. Importantly, these cells were negative for pan-cytokeratin (pan-CK), confirming the absence of epithelial cell contamination (Fig. 1C). These initial characterizations confirmed the successful establishment of patient-derived CAF cultures that retain key fibroblast markers.

### The transcriptomic landscape of PCa-derived primary CAFs reveals high heterogeneity and distinct subtypes

**Fig. 2:**
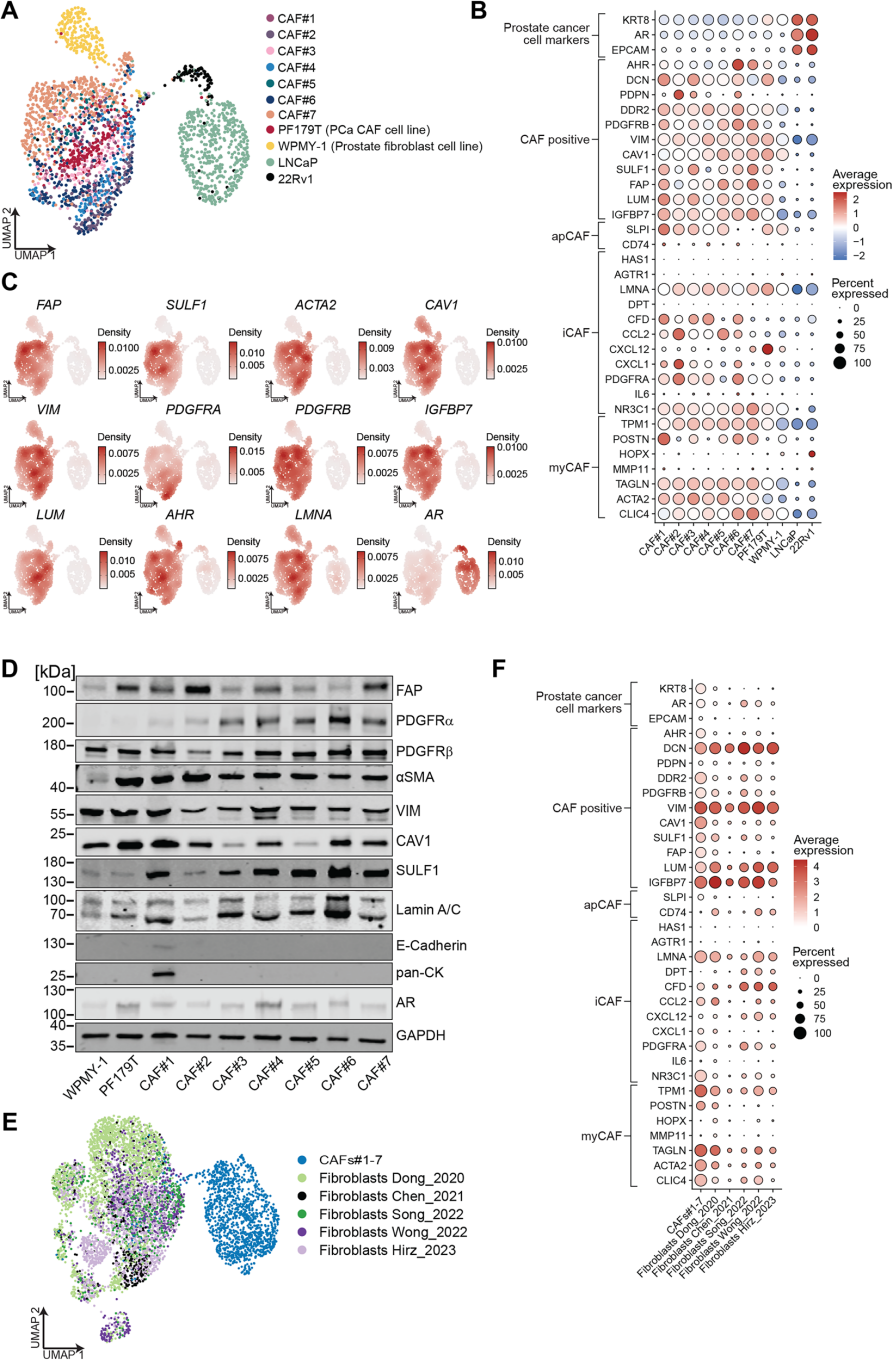
Single-cell transcriptomics reveal distinct features of ex vivo prostate CAFs. **A** Uniform Manifold Approximation and Projection (UMAP) of scRNA-seq data from ex vivo CAFs, two fibroblast cell lines, and two PCa cell lines colored by sample or cell line. **B** Dot plot showing the proportion of cells expressing selected marker genes (dot size) and expression intensity (color scale) across CAFs, fibroblast lines, and PCa cell lines. Values represent scaled averages of log-normalized counts. **C** Gene-weighted kernel density plots illustrating mRNA expression patterns of selected CAF and PCa markers, derived from log-normalized counts. **D** Western blot analysis validating protein-level expression of CAF- and PCa-associated markers in selected samples. **E** UMAP of CAFs integrated with publicly available fibroblast datasets. **F** Dot plot displaying the fraction of CAFs and reference fibroblasts expressing selected markers (dot size) and their relative expression levels (color shading), based on log-normalized count averages.

We performed scRNA-seq on the seven ex vivo CAF cultures, the established PCa CAF cell line PF179T, the benign prostate stromal cell line WPMY-1, and two PCa epithelial cell lines, LNCaP and 22Rv1. Uniform Manifold Approximation and Projection (UMAP) analysis revealed distinct clustering, with primary CAFs (CAF#1-7) generally grouping together, clearly segregated from WPMY-1 cells and the PCa epithelial cell lines (Fig. 2A). Furthermore, within the non-epithelial cell compartment, primary ex vivo CAFs formed a unique transcriptomic cluster, distinct from both PF179T and WPMY-1 fibroblast lines (Supplementary Fig. 2A), suggesting substantial divergence in their gene expression programs.

Analysis of established fibroblast lineage markers [25] showed generally high expression of *ACTA2* (αSMA) and *TAGLN* (transgelin) across most ex vivo CAF populations and PF179T, indicative of their activated, myofibroblastic state (Fig. 2B, C). However, canonical CAF markers such as *VIM* (vimentin), *FAP*, *SULF1*, and *CAV1* exhibited heterogeneous expression levels and percentages of expressing cells across the different CAF samples, reflecting intrinsic transcriptional diversity (Fig. 2B and Supplementary Fig. 2B). Stratification based on gene signatures for apCAFs (e.g., *SLPI*, *CD74*), iCAFs (e.g., *CFD*, *CCL2*, *CXCL12*, *PDGFRA*), and myCAFs (e.g., *TAGLN*, *ACTA2*) further highlighted significant inter-sample variability, underscoring the phenotypic heterogeneity within the primary CAF panel (Fig. 2B and Supplementary Fig. 2B). In contrast, PCa epithelial cells (LNCaP, 22Rv1) were characterized by high expression of *AR*, *EPCAM*, and *KRT8*, with a general absence of these stromal markers (Fig. 2B, C). Moreover, the expression of the immunoregulatory *NR3C1* gene and *CLIC4*, a TGFβ-regulated gene involved in myCAF differentiation, was specifically enhanced in CAFs, highlighting their importance in the stromal biology of PCa [26, 27]. Western blot analysis largely corroborated these scRNA-seq findings at the protein level, confirming positive FAP, PDGFRβ, αSMA, VIM, and CAV1 expression in the primary CAFs cell lysates (Fig. 2D).

To contextualize our ex vivo CAF models, we integrated their transcriptomic profiles with primary tissue fibroblasts from publicly available PCa datasets [28–31]. The ex vivo CAFs, including PF179T, formed a relatively distinct cluster from the in situ fibroblasts, likely reflecting culture-induced phenotypic shifts (Fig. 2E). Despite this, they retained strong expression of core stromal markers such as *DCN*, *VIM*, *LUM*, and *IGFBP7*, which were consistently observed across both ex vivo and in situ fibroblasts compared to the epithelial cancer cells (Fig. 2F and Supplementary Fig. 2C, D). This reinforces that while ex vivo culture modulates specific gene expression programs, our CAF models maintain fundamental fibroblast identity.

### Ex vivo CAFs show distinct stromal signaling pathways and lineage-specific transcription factor regulomes

**Fig. 3:**
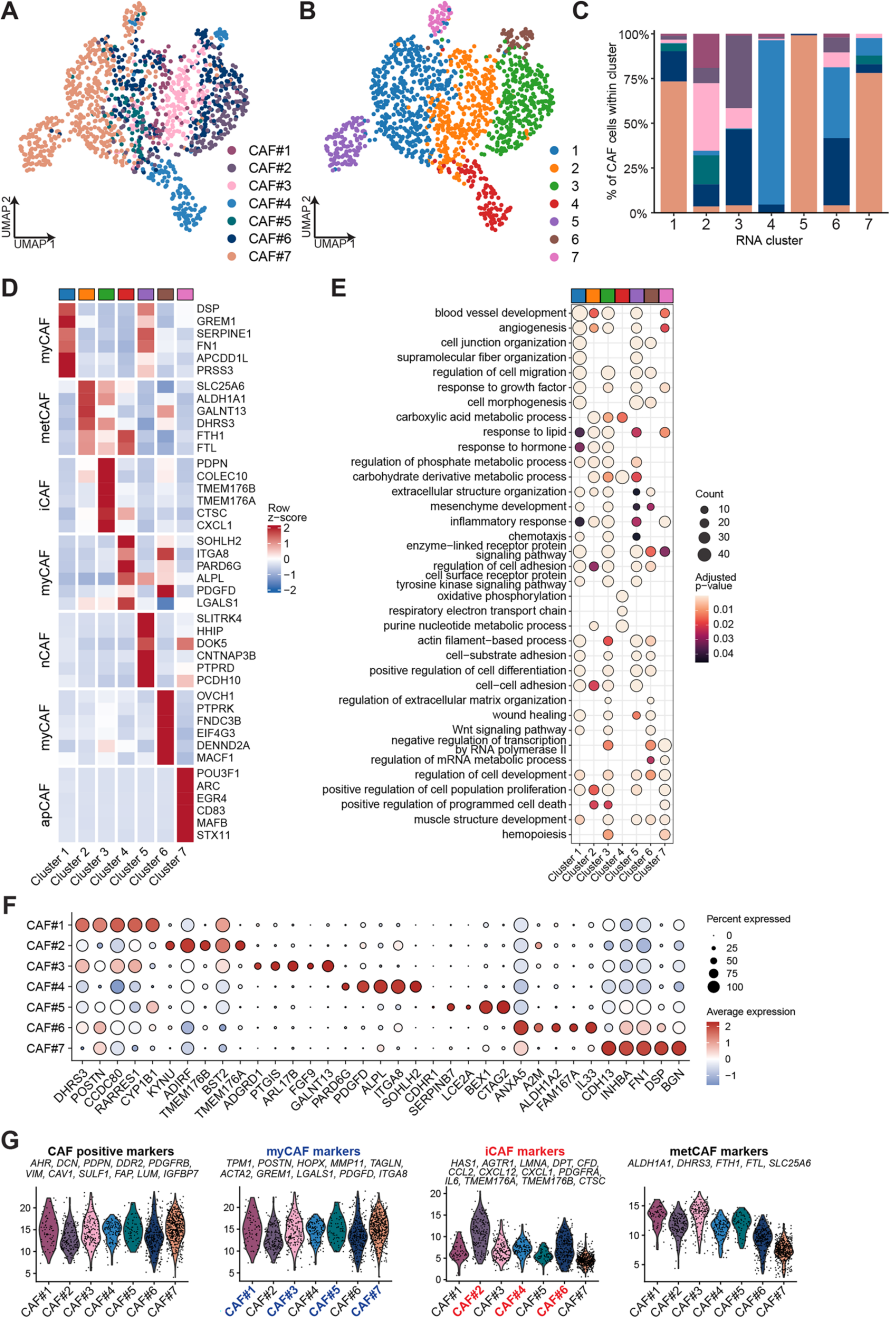
Transcriptomic heterogeneity of ex vivo CAFs. **A** UMAP of seven ex vivo CAFs colored by sample. **B** UMAP in (**A**) colored by assigned Leiden clusters. **C** Stacked bar chart showing the proportion (%) of cells from each sample within each Leiden cluster, colors correspond to (**A**). **D** Heatmap of the top six differentially expressed protein-coding genes per Leiden cluster. Values are cluster-average expression, row-scaled (z-scores), and capped at ±2. **E** Selected gene Ontology (GO:BP) enrichment results of the top 200 significant upregulated protein-coding genes in the Leiden clusters. Dot size reflects the number of genes in a term, and dot color indicates adjusted *p*-value. **F** Dot plot showing the scaled average expression of the top five significantly differentially expressed protein-coding markers for each CAF sample. Dot size represents the fraction of cells expressing the marker, while color intensity indicates the scaled average expression level. **G** Aggregated normalized expression of CAF-positive, myCAF, iCAF, and metCAF markers in CAFs.

To dissect the heterogeneity of ex vivo CAFs, we performed Leiden clustering of scRNA-seq data from all CAFs, which identified seven clusters, each represented by different proportions of CAFs (Fig. 3A–C). We then examined the top six differentially expressed genes in each cluster relative to all others and performed enrichment analysis to identify hallmark pathways (Fig. 3D, E). This analysis resolved five CAF subtypes: myCAFs, present across clusters 1, 4, and 6, with distinct emphases on ECM/contractility (cluster 1: *FN1*, *SERPINE*1, *GREM1*, *DSP*), adhesion (cluster 4: *ITGA8*, *LGALS1*), and cytoskeletal organization (cluster 6: *MACF1*, *FNDC3B*, *PTPRK*, *DENND2A*); iCAFs in cluster 3 (*CXCL1*, *TMEM176A/B*, *CTSC*); metabolic CAFs (metCAFs) in cluster 2 (*ALDH1A1*, *DHRS3*, *FTH1*, *FTL*, *SLC25A6*); apCAFs in cluster 7 (*CD83*); and neuronal-like CAFs (nCAFs) in cluster 5 (*SLITRK4*, *HHIP*, *DOK5*, *CNTNAP3B*, *PTPRD*, *PCDH10*).

We next examined the top five differentially expressed protein-coding genes across individual CAF models to capture functional diversity (Fig. 3F). Many of these markers encode ECM components (e.g., *FN1*, *COL11A1*, *POSTN*, *DSP*), signaling molecules (e.g., *PDGFD*, *GREM1*), immune modulators (*TMEM176A/B*), or stress-response and metabolic regulators (e.g., *FTH1*, *SERPINB7*, *ALDH1A2*), underscoring the diverse functional specializations of CAFs. Integrating these signatures, we found that CAF#1, CAF#3, CAF#5, and CAF#7 aligned most closely with a myCAF phenotype, whereas CAF#2, CAF#4, and CAF#6 showed stronger iCAF-like features. In addition, CAF#1 and CAF#3 exhibited enrichment for metCAF-associated markers (Fig. 3G). Together, these analyses demonstrate that the most upregulated genes in each CAF subtype are also the primary drivers of transcriptional segregation across the clusters, validating their role as key markers of CAF heterogeneity.

We then assessed intracellular signaling pathway activity using PROGENy [32]. This revealed that ex vivo CAFs and PF179T exhibited generally high activation of stromal-associated pathways, including JAK-STAT, TNFα, TRAIL, hypoxia, and TGFβ signaling (Supplementary Fig. 3A). These pathways are pivotal in CAF-driven inflammation, fibrosis, ECM remodeling, and angiogenesis. In contrast, NF-κB and androgen signaling were predominantly active in the epithelial LNCaP and 22Rv1 cell lines, consistent with their oncogenic roles. WPMY-1 cells showed a mixed but generally lower activation profile compared to CAFs.

To delineate regulatory programs, we applied pySCENIC [33] to infer transcription factor (TF) activity. This analysis identified distinct lineage-specific TF regulomes (Supplementary Fig. 3B). Ex vivo CAF models and PF179T showed significant upregulation of TF families like SOX (e.g., *SOX4*, *SOX7*), FOX (e.g., *FOXF1*, *FOXG1*), and *STAT3*, known to regulate ECM remodeling, inflammatory responses, and metabolic adaptation. WPMY-1 cells, while sharing some TFs, showed higher activity of TFs associated with cell cycle regulation and proliferation, such as E2F family members (*E2F1*, *E2F4*, *E2F8*) and *JUN*, highlighting key regulatory differences between tumor-derived CAFs and benign stromal fibroblasts.

### Therapeutically actionable CAF-associated genes in PCa

Our transcriptomic analysis identified several highly expressed CAF-associated genes, including *PDPN*, *PRSS3*, *GREM1*, and *LGALS1*, that represent either directly druggable targets or components of clinically actionable pathways, and we evaluated their relevance to the PCa stromal phenotype. PDPN (podoplanin), a transmembrane glycoprotein broadly expressed by CAFs across tumor types, has been effectively targeted in preclinical models using monoclonal antibodies and CAR-T approaches, resulting in robust anti-tumor activity [34]. PRSS3 (mesotrypsin), a serine protease upregulated in PCa CAFs and implicated in tumor invasion and metastatic dissemination, can be selectively inhibited by small-molecule compounds that suppress invasive growth in vitro [35]. GREM1 (gremlin-1), a secreted BMP antagonist, promotes angiogenesis and pro-tumorigenic signaling pathways, and neutralizing antibody strategies have demonstrated efficacy in preclinical fibrosis and cancer models [36]. Finally, LGALS1 (galectin-1), a lectin secreted by CAFs that drives immune evasion by binding to glycosylated receptors on T cells and other immune subsets, is pharmacologically tractable, with small-molecule inhibitors currently under investigation [37]. Along these new targets, we also investigated the relevance of FAP expression, a canonical stromal marker linked to ECM remodeling, angiogenesis, EMT, and immunosuppression leading to poor PCa prognosis [38].

**Fig. 4:**
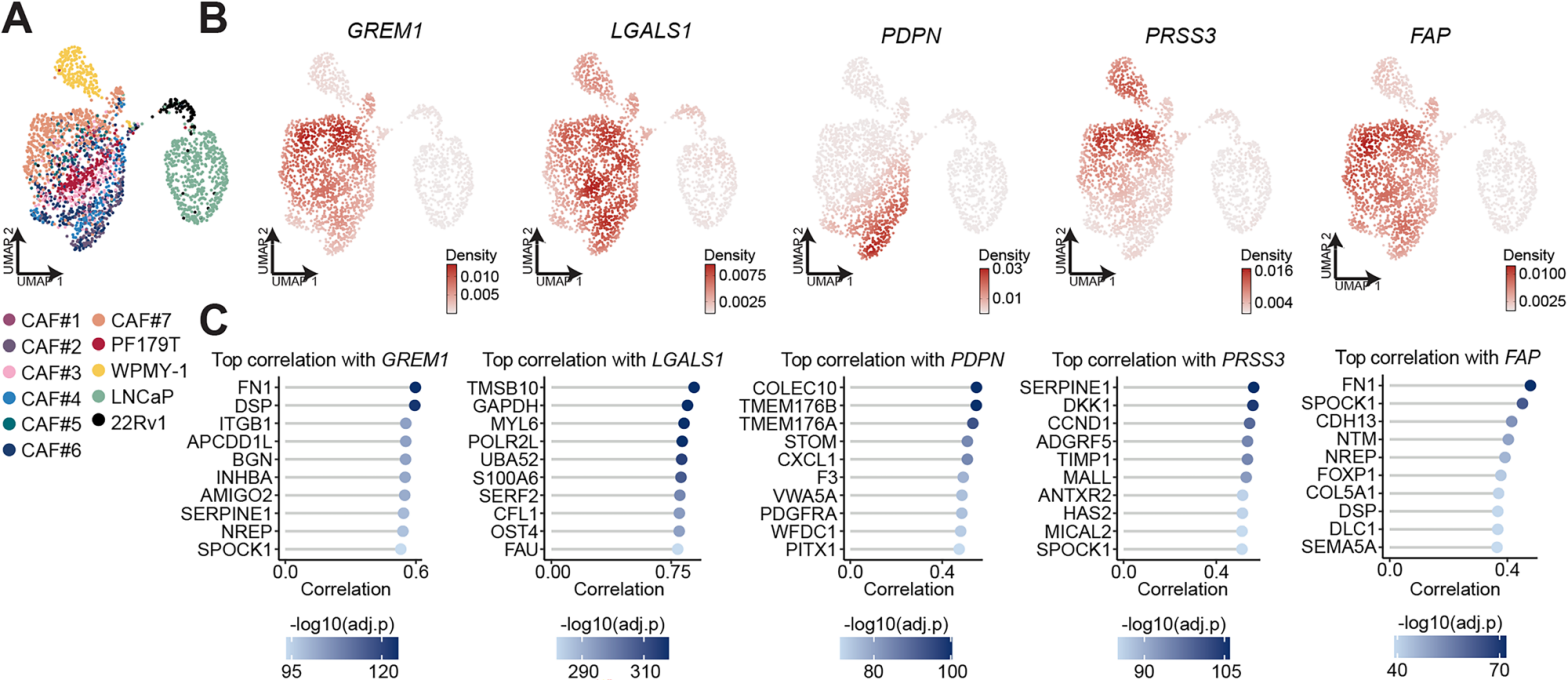
Marker expression and co-expression across ex vivo CAFs. **A** UMAP of scRNA-seq data from ex vivo CAFs, two fibroblast cell lines, and two PCa cell lines colored by sample or cell line. **B** Gene-weighted kernel density plots illustrating mRNA expression patterns of *GREM1*, *LGALS1*, *PDPN*, *PRSS3*, and *FAP*, derived from log-normalized counts. **C** Lollipop plots showing the top 10 significant positively correlated protein-coding genes with *GREM1*, *LGALS1*, *PDPN, PRSS3*, and *FAP*. Pearson correlation values are shown on the x-axis, with genes ordered by correlation strength. Color shading represents the significance level, with darker shades indicating stronger significance.

Transcriptomic analysis showed broader expression of *GREM1*, *LGALS1*, and *FAP* across ex vivo CAFs, whereas *PDPN* and *PRSS3* displayed more cluster-specific patterns, suggesting specialized roles (Fig. 4A, B). In public datasets, *GREM1*, *LGALS1*, and *FAP* were largely CAF-restricted, while *PDPN* and *PRSS3* were also detected in epithelial clusters, indicating functions beyond stroma (Supplementary Fig. 4A, B). Pearson correlation analysis revealed distinct stromal programs associated with each CAF target (Fig. 4C). *GREM1* was linked to integrin/ECM signaling (*FN1*, *ITGB1*, *BGN*) and pro-angiogenic factors (*INHBA*, *SERPINE1*), consistent with roles in remodeling and vascularization. *LGALS1* correlated with cytoskeletal and metabolic regulators (*CFL1*, *S100A6*, *GAPDH*), reflecting enhanced motility and secretory activity. *PDPN* was associated with immune and angiogenic mediators (*CXCL1*, *TMEM176A/B*, *PDGFRA*) and coagulation signaling (*F3*), supporting its role in inflammatory crosstalk. *PRSS3* correlated with ECM remodeling and Wnt pathway regulators (*SERPINE1*, *TIMP1*, *HAS2*, *DKK1*, *CCND1*), linking it to proliferative and invasive behavior. Finally, *FAP* was associated with ECM and adhesion genes (*FN1*, *COL5A1*, *SPOCK1*, *CDH13*) and regulators of migration (*NREP*, *SEMA5A*), consolidating its central role in matrix deposition and tumor invasion, as previously shown [38]. Together, these profiles highlight complementary tumor-supportive programs in PCa CAFs and underscore the therapeutic potential of targeting these genes to disrupt stromal remodeling, angiogenesis, and immune evasion in PCa.

### Primary CAFs exhibit shared drug response phenotypes

**Fig. 5:**
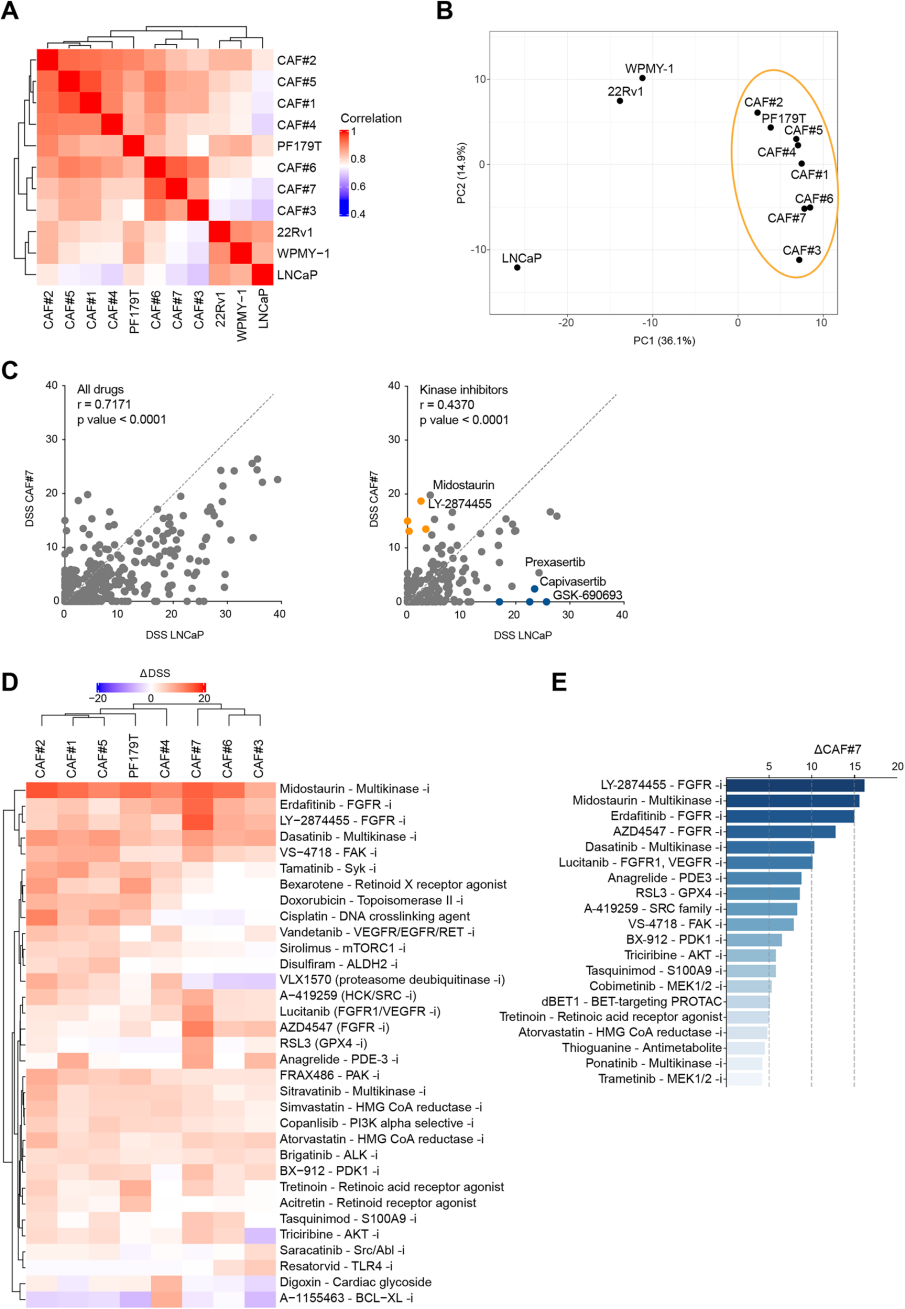
Ex vivo CAFs display distinct drug sensitivity profiles with selective vulnerability to kinase inhibitors. **A** Heatmap of pairwise Pearson correlations of DSS profiles across 396 compounds shows clustering of primary CAFs (#1–7) with the established CAF cell line PF179T, distinct from WPMY-1, LNCaP, and 22Rv1. **B** Principal component analysis (PCA) of DSS profiles confirms separation of CAFs from epithelial and benign stromal cell lines. **C** Scatterplots comparing drug response profiles between CAF#7 and LNCaP across all compounds (left) and kinase inhibitors (right) highlight drugs with preferential activity in CAFs, including FGFR and multikinase inhibitors. **D** Heatmap of top ΔDSS compounds per sample reveals heterogeneous but recurrent CAF-selective responses, including inhibitors of FGFR, FAK (VS-4718), multikinases (dasatinib, midostaurin), and stress-response pathways (e.g., ferroptosis inducer RSL3, PDK1 inhibitor BX-912). **E** Top-ranked ΔDSS compounds in CAF#7 relative to LNCaP include FGFR inhibitors, multikinase inhibitors, and non-canonical targets such as ferroptosis, PDK1, and retinoid signaling pathways.

To characterize the drug response landscape of primary CAFs, we performed high-throughput DSRT using a panel of 396 oncology compounds, including FDA-approved drugs and investigational compounds (Supplementary Fig. 5A and Supplementary Tables 2 and 3). Pairwise correlation analysis of drug sensitivity scores (DSS) revealed a strong concordance among primary CAFs (#1–7) and PF179T, forming a distinct cluster that was clearly separated from the benign stromal line WPMY-1 and the PCa cell lines LNCaP and 22Rv1 (Fig. 5A). Principal component analysis (PCA) further supported this separation, with the CAFs and PF179T clustering tightly and distinctly from epithelial and benign stromal lines (Fig. 5B), indicating a shared drug response phenotype among CAFs.

Comparing CAF#7 (a FAP-high, myCAF subtype) with LNCaP cells, we observed moderate overall DSS correlation (*r* = 0.7171 all drugs; *r* = 0.4370 kinase inhibitors), with several compounds showing markedly higher sensitivity in CAF#7 (Fig. 5C). These included multikinase inhibitors like midostaurin and the FGFR inhibitor LY-2874455. Conversely, AKT pathway inhibitors (capivasertib, GSK-690693) were more active in LNCaP, due to constitutively active AKT signaling resulting from PTEN loss.

A heatmap of the top differentially active compounds revealed a subgroup of drugs with consistent activity across the CAF cohort, including midostaurin, dasatinib, VS-4718, and FGFR inhibitors (Fig. 5D). Despite inter-sample variability, these compounds showed reproducible activity in multiple CAF ex vivo samples. Quantification of differential DSS (ΔDSS) between CAF#7 and LNCaP highlighted several kinase inhibitors as top-ranking compounds, further supporting selective CAF vulnerabilities to kinase-targeted therapies (Fig. 5E).

The identification of compounds with selective activity in CAFs - including FGFR inhibitors (erdafitinib, LY-2874455), the multikinase inhibitor midostaurin, the FAK inhibitor VS-4718, and less canonical agents such as the ferroptosis inducer RSL3 and the PDK1 inhibitor BX-912—underscores the potential of targeting the TME as a complementary strategy in PCa therapy (Supplementary Fig. 5B and Supplementary Table 4). These findings highlight the functional heterogeneity of the tumor stroma and support further exploration of CAF-directed therapeutic approaches.

## Discussion

This study successfully established and comprehensively characterized a panel of seven primary ex vivo PCa CAF models, providing a resource that captures the inherent heterogeneity of the tumor stroma. By integrating IHC, scRNA-seq, and DSRT, we have unveiled distinct CAF subtypes, each with unique molecular signatures, pathway activities, and, crucially, subtype-aligned drug vulnerabilities.

Altogether, our analysis resolved five CAF subtypes: myCAFs defined by ECM, adhesion, and cytoskeletal genes; iCAFs enriched for chemokines and immune regulators; metCAFs characterized by retinoid, iron, and mitochondrial metabolism; apCAFs with immune-regulatory and antigen-presenting markers; and nCAFs expressing neuronal adhesion and Hedgehog pathway genes. These findings have significant implications for understanding CAF biology in PCa and developing more effective stromal-targeted therapies.

A major challenge in targeting CAFs is their dynamic adaptability; selective depletion of one subtype may trigger compensatory activation of others. This plasticity may explain why FAP-targeted therapies have demonstrated limited success in clinical trials. Rather than eradicating CAFs, a more promising approach may involve modulating their tumor-supportive functions. Our findings expand the repertoire of therapeutically actionable stromal drivers in PCa, underscoring that CAFs not only remodel the ECM but also orchestrate angiogenesis, immune evasion, and invasive signaling through distinct gene programs. The druggability of PDPN, PRSS3, GREM1, and LGALS1, alongside the established relevance of FAP, provides multiple entry points for stromal-directed interventions that could complement tumor cell–targeted therapies. Clinically, targeting these CAF-associated pathways may attenuate stromal support for tumor progression, overcome microenvironment-mediated therapy resistance, and open new avenues for precision stromal targeting in PCa.

Our findings also suggest that TKIs and FGFR inhibitors could impair CAF signaling networks, offering potential for combination therapy. However, despite these insights, several limitations must be addressed. The use of 2D cell culture models does not fully capture the complexity of the in vivo TME, where CAF-cancer cell interactions, hypoxia, and mechanical forces drive CAF function and plasticity. Additionally, the limited sample size (*n* = 7) underlines the need for larger patient cohorts to validate these findings. Our drug screening approach focused on bulk cell viability, which does not account for CAF subpopulation dynamics and adaptive responses. Future studies should integrate 3D co-culture models and single-cell functional assays to further dissect CAF plasticity and drug resistance mechanisms.

Beyond PCa, our findings contribute to the broader understanding of CAF heterogeneity in solid tumors, where similar pro-invasive, immune-modulatory, and fibrotic CAF subsets have been described in pancreatic, breast, and lung cancers. The conserved pro-tumorigenic roles of CAFs suggest that stromal-targeted therapies could have broader clinical applications beyond PCa.

## Materials and Methods

Detailed methods can be found in the Supplementary material.

### Histological and IHC analysis

FFPE tissue sections (3.5 μm) were stained with H&E and antibodies against FAP and PDGFRβ. Slides were scanned digitally and reviewed by a certified pathologist. Tumor content, epithelial-to-stromal ratio, and FAP expression were visually assessed, with FAP scored semi-quantitatively based on staining intensity and extent.

### Cell culture

Fresh prostate tumor tissue was enzymatically dissociated using Miltenyi’s dissociation kit according to the manufacturer’s protocol (Miltenyi Biotec, Bergisch Gladbach, Germany). Fibroblasts were cultured at low-passage (p4–8) for the experiments. PCa cell lines and control fibroblast lines were obtained from ATCC and cultured as instructed.

### Drug sensitivity and resistance testing (DSRT) screens

DSRT was performed on seven primary CAFs, along with PF179T, WPMY-1, LNCaP, and 22Rv1 cell lines using an oncology compound library comprising 396 approved and investigational drugs, as previously described [39].

### scRNA-seq data analysis

ScRNA-seq was performed using the 10× Genomics 3′ v3 platform with feature barcoding, sequenced on an Illumina NovaSeq 6000, as previously described [40]. Data were analyzed in R (4.3.2) with Seurat (5.0.3) for pre-processing, normalization, Harmony integration, and clustering. CAF subtypes were characterized through clustering, differential gene expression analysis, pySCENIC regulome analysis, correlation, enrichment, and pathway activity scoring (decoupleR/PROGENy). Publicly available scRNA-seq datasets from five PCa studies (GEO: GSE137829, GSE141445, GSE176031, GSE185344, and GSE181294) were individually processed, integrated, and annotated with scType.

## Supplementary information

Supplementary Figure 5

Supplementary Materials and Methods

Supplementary Figure 1

Supplementary Figure 2

Supplementary Figure 3

Supplementary Figure 4

Supplementary Table 1

Supplementary Table 2

Supplementary Table 3

Supplementary Table 4

Figure source data

Uncropped Western Blots

## Data Availability

The raw counts data and corresponding metadata for all samples generated and analyzed in this study, together with the Supplementary Source Data, are available in Mendeley Data (10.17632/g3pvkrpr2k.1). The DSRT DSSs scores are available in Supplementary Table 2. The public datasets are available at GEO under their respective accession numbers. All original code has been deposited at GitHub (https://github.com/ungureanulab/CAF-profiling).
