# Supplementary figures and images for: Molecular profiling of ex vivo prostate cancer CAF models captures stromal heterogeneity and drug vulnerabilities
Source: Cell Death Discov. 2025 Nov 6;11:507. doi: 10.1038/s41420-025-02792-3 (PMC12592484; doi:10.1038/s41420-025-02792-3)

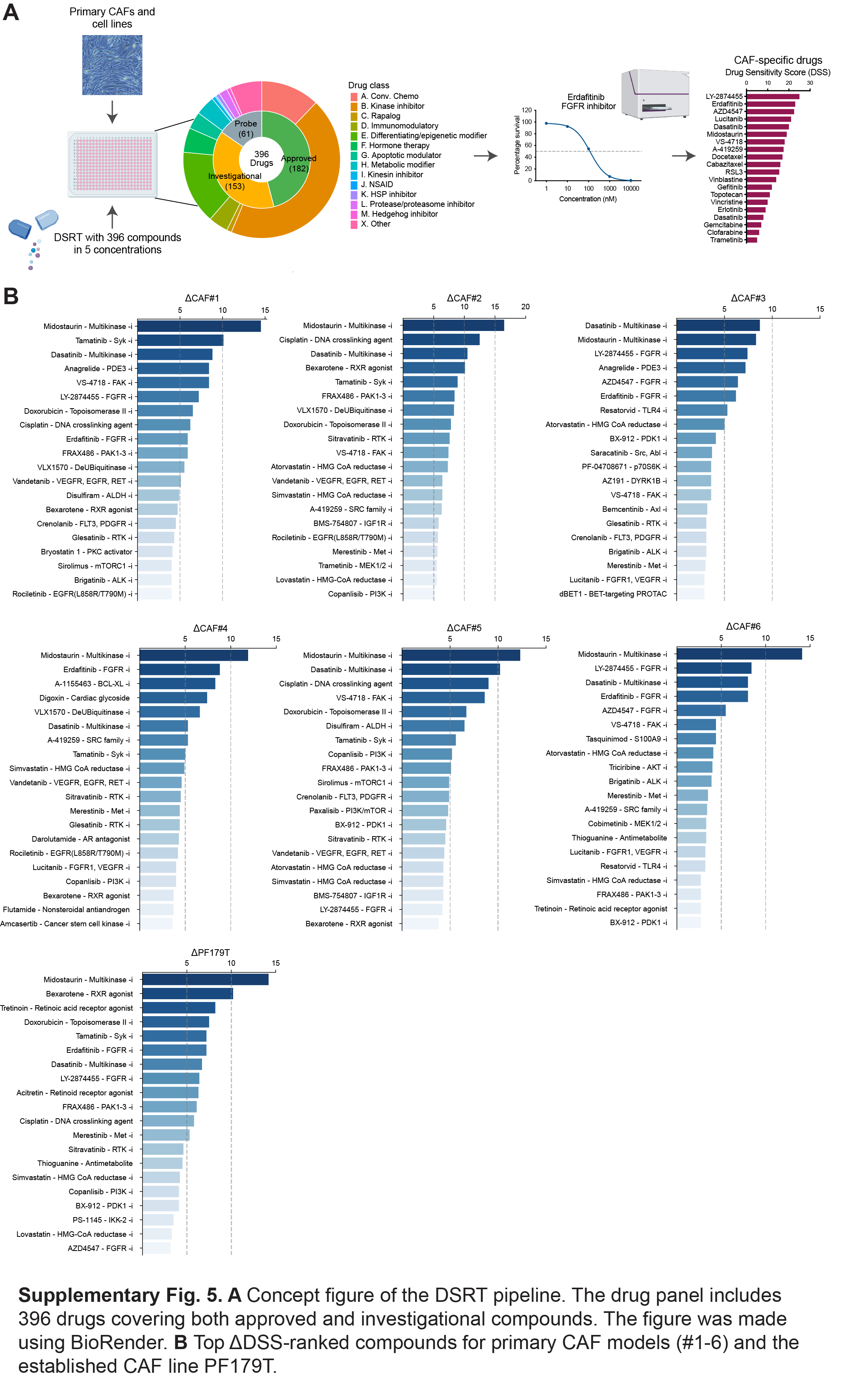

Supplement: Supplementary file 1 — Supplementary Figure 5 [file 41420_2025_2792_MOESM1_ESM.png]

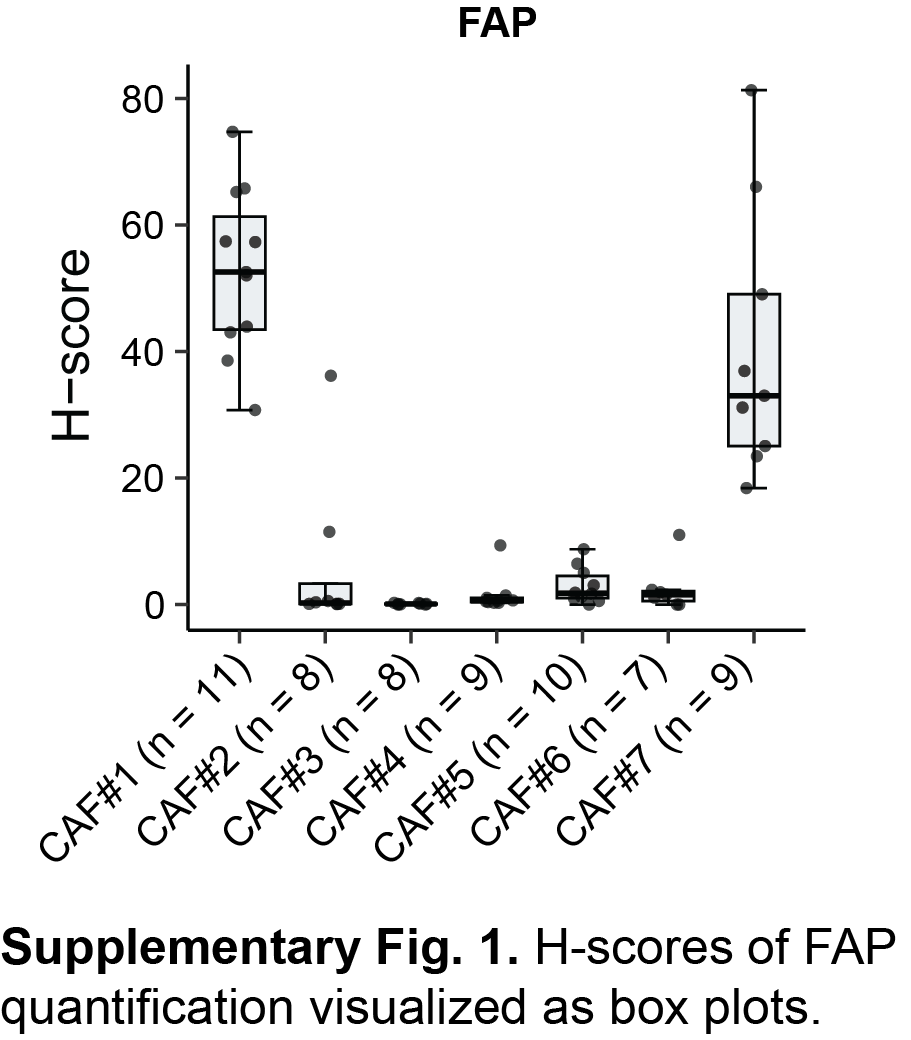

Supplement: Supplementary file 3 — Supplementary Figure 1 [file 41420_2025_2792_MOESM3_ESM.png]

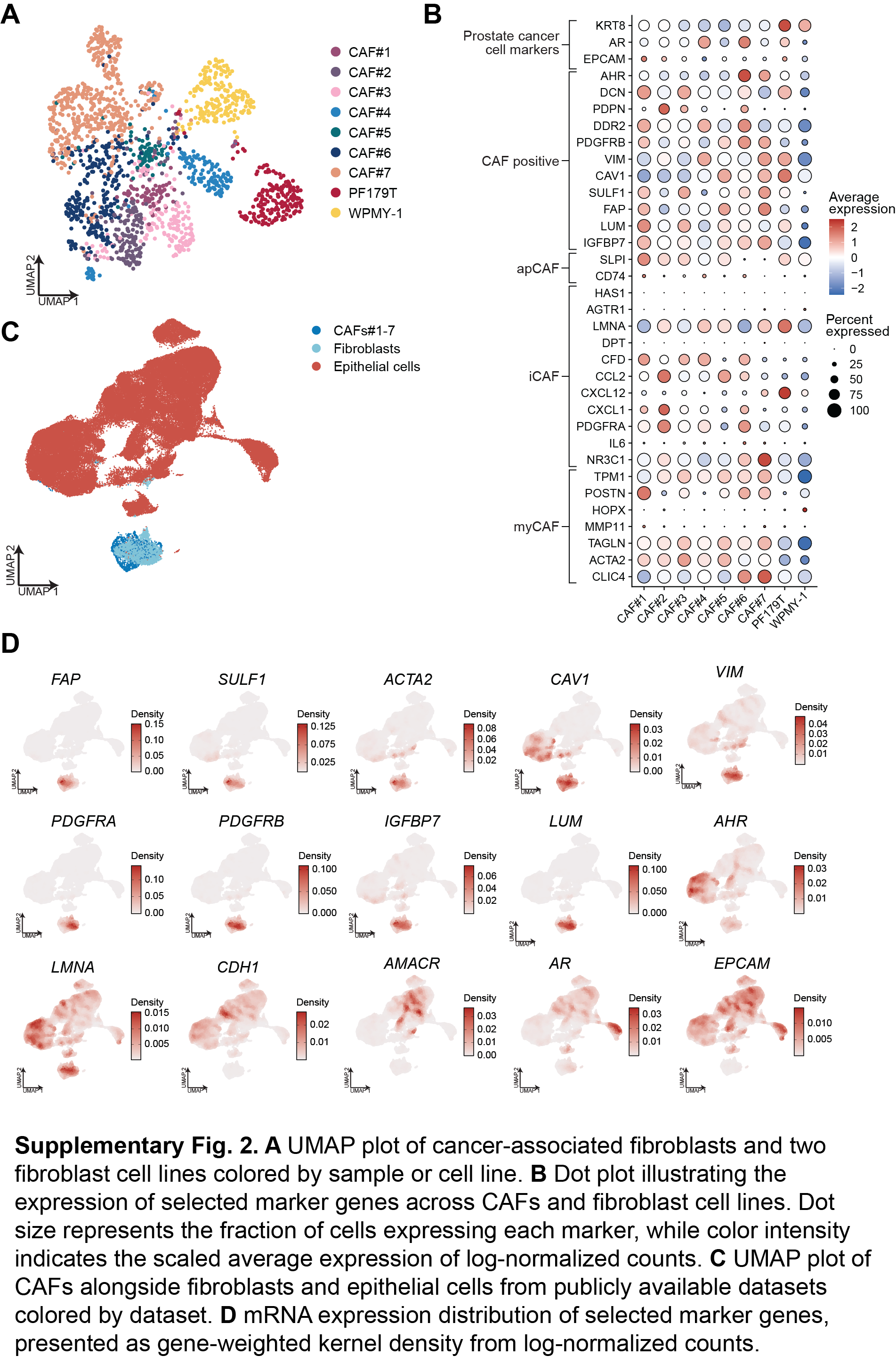

Supplement: Supplementary file 4 — Supplementary Figure 2 [file 41420_2025_2792_MOESM4_ESM.png]

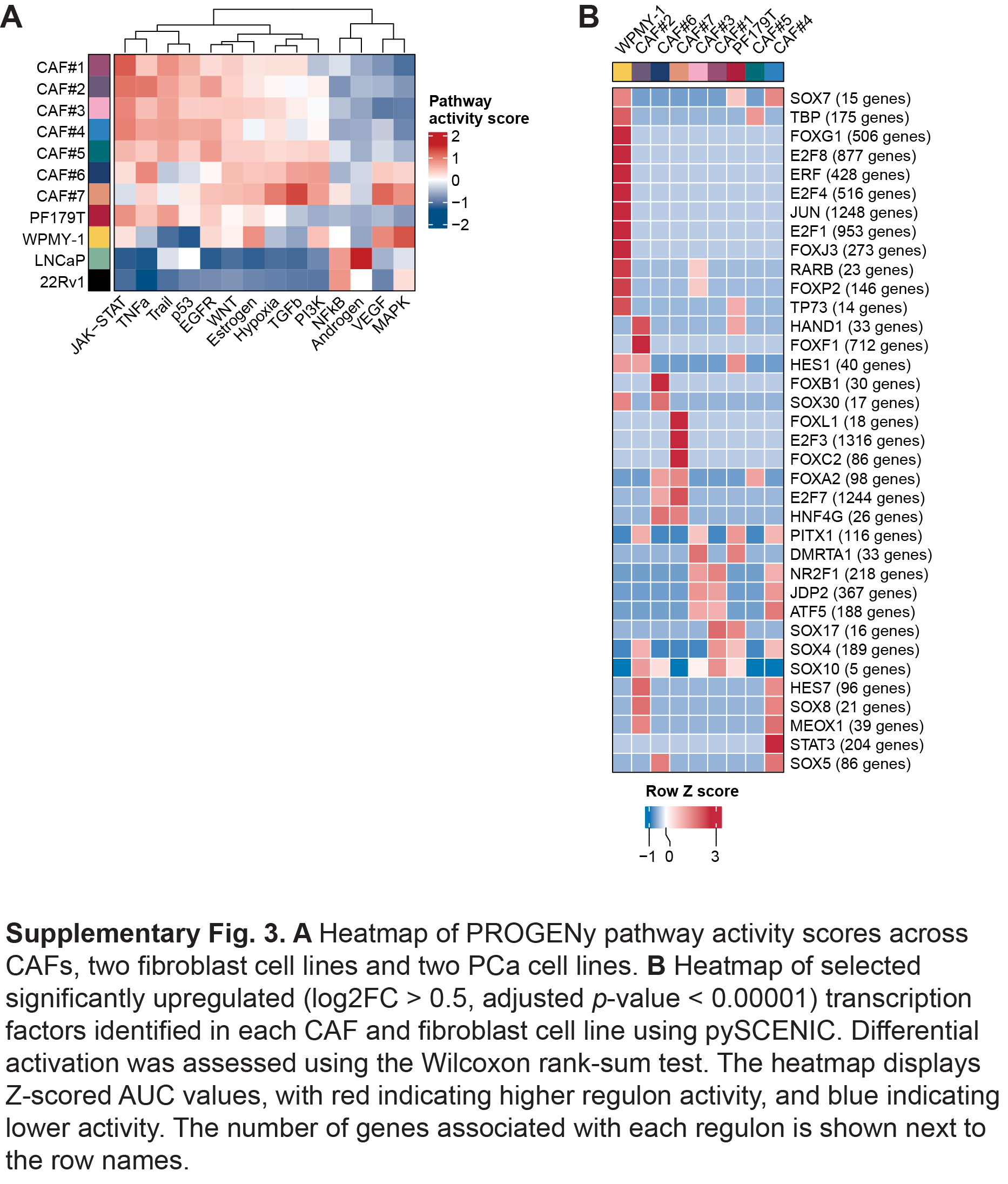

Supplement: Supplementary file 5 — Supplementary Figure 3 [file 41420_2025_2792_MOESM5_ESM.png]

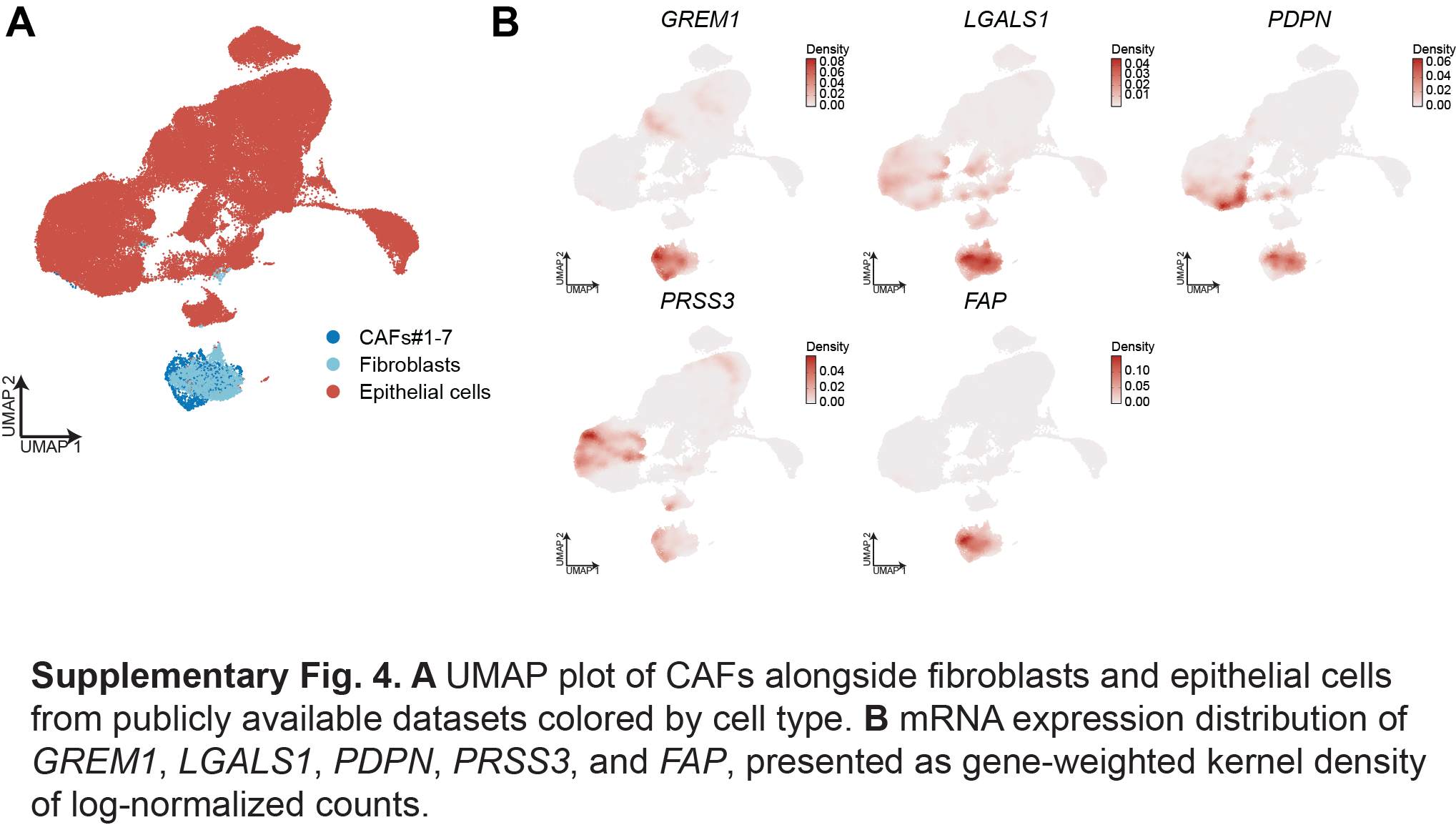

Supplement: Supplementary file 6 — Supplementary Figure 4 [file 41420_2025_2792_MOESM6_ESM.png]

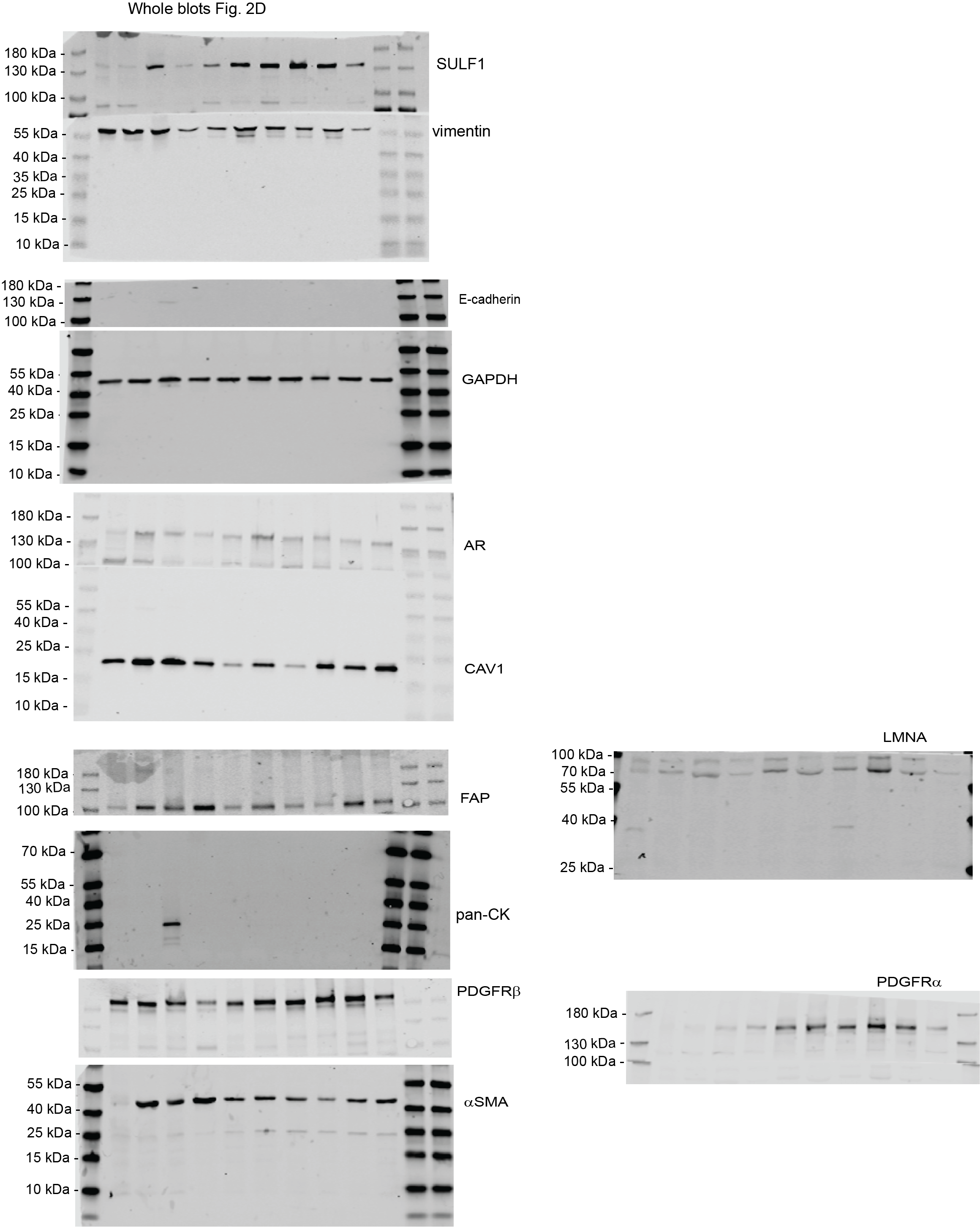

Supplement: Supplementary file 12 — Uncropped Western Blots [file 41420_2025_2792_MOESM12_ESM.png]
