# Supplementary material for: Molecular profiling of ex vivo prostate cancer CAF models captures stromal heterogeneity and drug vulnerabilities
Source: Cell Death Discov. 2025 Nov 6;11:507. doi: 10.1038/s41420-025-02792-3 (PMC12592484; doi:10.1038/s41420-025-02792-3)
Supplement: Supplementary file 2 — Supplementary Materials and Methods [file 41420_2025_2792_MOESM2_ESM.docx]

**Molecular Profiling of Ex Vivo Prostate Cancer CAF Models Captures Stromal Heterogeneity and Drug Vulnerabilities**

Frida Rantanen^†^, Astrid Murumägi^†^, Mariliina Arjama, Katja Välimäki, Elina Multamäki, Tuomas Mirtti, Antti Rannikko, Teijo Pellinen, Daniela Ungureanu*, Olli Kallioniemi*

† These authors contributed equally to this work.

*Corresponding authors.

**Supplementary material**

**Materials and methods**

**Sample collection**

Tumor biopsies were collected from seven prostate cancer patients undergoing prostatectomy at Helsinki University Central Hospital. The sample collection was conducted under Ethics Committee approval (HUS/850/2017 and HUS/155/2021).

**Tissue processing and establishment of PCa-derived cancer-associated fibroblasts**

Fresh tumor tissue was transported from the hospital to the laboratory on ice in HBSS buffer and processed into a single-cell suspension using Miltenyi’s tumor dissociation kit and GentleMacs Dissociator (Miltenyi Biotec). Red blood cells were removed using Miltenyi’s lysis solution according to the manufacturer’s protocol. Cancer-associated fibroblasts (CAFs) were established by plating the resulting cell suspension in culture conditions favoring fibroblast growth. After a few days of culture, fibroblasts exhibited rapid adherence and proliferation, gradually outgrowing other cell types. Primary CAFs were maintained in Advanced RPMI-1640 medium supplemented with 10% FBS, 1% penicillin-streptomycin and Primocin (Invivogen). Low-passage (p4–8) CAFs were used in all experiments.

**Cell Lines**

Human prostate cancer cell lines LNCaP (CRL-1740) and 22Rv1 (CRL-2505), as well as the hTERT-immortalized prostate CAF cell line PF179T (CRL-3290) and the myofibroblast cell line WPMY-1 (CRL-2854), were obtained from the American Type Culture Collection (ATCC). All cells were cultured in RPMI-1640 medium supplemented with 10% FBS, 1% penicillin-streptomycin at 37°C in a humidified incubator containing 5% CO2.

**Immunohistochemistry (IHC) and histopathological assessment**

Formalin-fixed, paraffin-embedded (FFPE) tissue blocks were cut into 3.5 μm sections and stained with hematoxylin and eosin (H&E). IHC staining was performed using anti-FAP (1:500, ab207178, Abcam) and anti-PDGFRβ (1:100, CST#3169, Cell Signaling Technology) antibodies following standard protocols. Stained sections were scanned at high resolution using a whole-slide scanner (Pannoramic 250 Flash III, 3DHISTECH) with a 20× objective. H&E-stained sections and FAP IHC-stained slides were reviewed by co-author Professor Tuomas Mirtti, a certified pathologist. Tumor content percentage, epithelial-to-stromal ratio, and FAP expression levels were visually estimated based on morphological evaluation under a light microscope. Tumor content was assessed as the approximate proportion of tumor cells relative to the total tissue area, while the epithelial-to-stromal ratio was determined based on the observed cellular distribution. FAP expression was semi-quantitatively evaluated using a categorical scale (+/-, +, ++, +++), reflecting the intensity and extent of staining within the tumor microenvironment.

**Image analysis with QuPath**

Digital images were analyzed using QuPath (0.5.1) (1). Individual cells were detected using QuPath’s built-in *Positive Cell Detection* module, which segments nuclei based on hematoxylin staining and then estimates the extent of marker expression in the cytoplasm or nucleus, depending on the staining pattern. Detection parameters (e.g., nucleus size, thresholding, and background correction) were optimized for our dataset to ensure accurate segmentation of both positive and negative cells. For each region of interest (ROI), the algorithm classified cells according to staining intensity (negative, weak, moderate, strong). The output was used to calculate H-scores, defined as:

H-score=(0×%negative)+(1×%weak)+(2×%moderate)+(3×%strong) resulting in a continuous score from 0 to 300.

Quantification was performed across all annotated ROIs, and summary statistics were exported from QuPath for downstream analysis. The resulting H-scores of FAP were visualized as box plots using ggplot2 (3.5.1) (2).

**Immunofluorescence Assay**

Primary CAFs and cell lines were seeded in 384-well cell culture plates (Corning) and cultured for 72 hours. The cells were fixed with 4% paraformaldehyde (PFA), blocked, and incubated overnight with the following primary antibodies: FAP (1:200, #2070718, Abcam), PDGFRβ (1:100, #3169, CST), AR-H280 (1:200, #sc-13062, Santa Cruz Biotechnology), pan-cytokeratin (Pan-CK; 1:200, ab7753, Abcam), vimentin (1:200, ab92547, Abcam) and α-Sma (1:200, #8717). Following primary incubation, cells were incubated with Phalloidin AF647 (#A22287, Thermo Fisher Scientific), fluorescence-conjugated secondary antibodies, and Hoechst 33342 (Merck). Imaging was conducted using a 20x objective on the PerkinElmer Opera Phenix at the FIMM High Content Imaging and Analysis Unit.

**Drug Sensitivity and Resistance Testing (DSRT)**

DSRT was performed on seven primary CAFs, along with PF179T, WPMY-1, LNCaP, and 22Rv1 cell lines using an oncology compound library comprising 396 approved and investigational drugs, as previously described (3). The list of compounds included in the library, together with their tested concentration ranges, drug mechanisms/targets, and classification, is provided in Supplementary Table 2. Briefly*,* cells were dispensed into 384-well plates pre-printed with compounds at five concentrations spanning a 10,000-fold range. DMSO and benzethonium chloride (BzCl) served as negative and positive controls, respectively. After 72 hours of incubation, cell viability was measured using the CellTiter-Glo 2.0 assay (Promega), and luminescence was detected with a PHERAstar FS plate reader (BMG Labtech). Dose-response curves were generated, and drug sensitivity scores (DSS) were calculated using the in-house BREEZE analysis pipeline (breeze.fimm.fi) (4,5). Briefly, DSS quantifies compound response by calculating a partial area under the dose–response curve (AUC, 10–100% activity window), and a dose-window either from the minimum concentration tested or from the concentration where the %inhibition reaches 10%. The DSS2 metric was applied, which normalizes the partial AUC by the logarithm of the upper asymptote of the logistic curve. Higher DSS values reflect greater compound sensitivity. DSS has been shown to be reproducible across screening sites and superior to conventional metrics such as IC50 or activity area (6). To compare drug responses between CAFs and LNCaP cell line, ΔDSS was calculated by subtracting the LNCaP DSS from each CAF DSS individually (Supplementary Table 4).

**Immunoblotting**

Cells were lysed with a Triton X-based lysis buffer containing 50 mM Tris-HCl (pH 7.5), 10% glycerol, 150 mM NaCl, 1 mM EDTA, 1% Triton X-100 (X100-100ML, Sigma-Aldrich), 50 mM NaF, and supplemented with protease and phosphatase inhibitors. The inhibitors used were Phosphatase Inhibitor Cocktail (2 tubes, 100×; B15001) and Protease Inhibitor Cocktail (EDTA-free, 100× in DMSO; B14001), both sourced from Bimake (now Selleck Chemicals). Lysates were mixed with Laemmli sample buffer (Bio-Rad Laboratories, California, USA), separated by SDS-PAGE, and transferred to nitrocellulose membranes. Membranes were then incubated with the following primary antibodies (used at a 1:500 dilution, unless otherwise stated): αSMA (#M0851, Dako), AR-H280 (#sc-13062, Santa Cruz Biotechnology), Caveolin-1 (#3267, Cell Signaling Technology (CST)), E-cadherin (#BD610182, BD Biosciences), FAP (#207178, Abcam), GAPDH (#G8795, Sigma-Aldrich, 1:1000), Pan-CK (#MA5-13156, Invitrogen), PDGF Receptor β (#3169, CST), SULF1 (#PA-115984, Invitrogen), and Vimentin (#5741, CST). Secondary antibodies used were IRDye® 680RD Goat anti-Mouse IgG and IRDye® 800CW Goat anti-Rabbit IgG (LI-COR, Lincoln, NE, USA). Blots were scanned using the Odyssey® Fc Imaging System (LI-COR), and image analysis was performed with Image Studio Lite Ver 5.2 software (LI-COR).

**Single-cell RNA Sequencing (scRNA-seq)**

Library Chemistry, Sequencing, and Preprocessing of Raw FASTQ Files

Sample preparation for scRNA-seq was done as previously published (7). Single-cell gene expression profiling was performed using the 10x Genomics Chromium Single Cell 3’ RNA-seq platform (10x Genomics, #CG000317). The Chromium Single Cell 3’RNAseq workflow, including library preparation, were done using the Chromium Next GEM Single Cell 3’ Gene expression version 3 Dual Index chemistry with Feature Barcoding technology. Sequencing of libraries was performed with Illumina NovaSeq 6000. Preprocessing of FASTQ files was carried out using the Cell Ranger pipeline (8.0.0, 10x Genomics). Cellranger mkfastq was used to generate FASTQ files, while cellranger count performed alignment and UMI quantification. Reads were aligned to the GRCh38 reference genome (GENCODE v32/Ensembl 98) (8).

scRNA-seq Data Analysis

The scRNA-seq data analyses were performed as previously described (7) using R (4.3.2) (9). The pre-processing was done using the R package Seurat (5.0.3) (10). First, the hashtag-oligo (HTO) data from the experimental batches was normalized using the NormalizeData function. Cells were demultiplexed with the HTODemux function using default settings, and any negative or doublet-classified cells were removed. The remaining HTO-classified cells underwent quality control, which involved removing cells with too many or too few counts, as well as cells with too high mitochondrial counts. CAFs were further filtered to remove cells with higher than mean mRNA counts of *AR*, *CDH1* and *EPCAM*.

The demultiplexing was followed by normalization and variance stabilization using the NormalizeData function, and highly variable genes were identified with FindVariableFeatures using default settings. The data layers were merged, and cell cycle heterogeneity was assessed with CellCycleScoring, using the cell cycle phase references provided by Tirosh et al. (11). Next, the Seurat object was split back into its original layers, and the data was scaled using ScaleData while regressing out G2M and S phase scores. Principal component analysis (PCA) was conducted with RunPCA using default parameters, and experimental batches were integrated using Harmony (1.2.0) (12) via the IntegrateLayers function. The number of dimensions for downstream analyses was determined with ElbowPlot. Using the harmony-corrected embedding and these dimensions, RunUMAP was computed, after which a k-nearest-neighbor graph was built with FindNeighbors. Clustering was then performed with FindClusters using the Leiden algorithm (13) at a resolution of 0.3. Leiden cluster composition bar charts were generated using ggplot2. CAF counts were aggregated by RNA cluster and sample and expressed as within-cluster percentages. Markers for Leiden clusters were identified using FindAllMarkers (test.use = ”wilcox”, layer = ”data”, only.pos = TRUE). The results were filtered to contain only protein-coding significant (adjusted *p*-value < 0.05) results. Top six genes per cluster were visualized as a heatmap of cluster-average expression, scaled per gene (row-wise z-score) across clusters and capped at ±2, using ComplexHeatmap (2.15.4) (14). For each Leiden cluster, significant upregulated markers (avg_log2FC > 0.5, adjusted *p*-value < 0.05) were ranked by adjusted *p*-value and the top 200 genes were used for GO:BP enrichment using the gost() function of the gprofiler2 package (0.2.3) (15). The enriched terms were filtered to intersection >= 4 genes and term size < 1000. Results were compiled across clusters and selected processes were visualized as a dot plot using ggplot2. Differential gene expression analysis of each CAF was performed using the FindAllMarkers function and the Wilcoxon Rank Sum test, extracting only positive results. The average expression of the resulting top five most significant protein coding genes for each CAF was visualized using the DotPlot function. Aggregated normalized counts of CAF-positive, metCAF, myCAF, and iCAF markers in CAFs were visualized in violin plots using the VlnPlot function.

The kernel density estimates of relevant genes were visualized using the Nebulosa package (1.12.1) (16) using the normalized data slot. Average expression of relevant fibroblast (17) and cancer cell marker genes were visualized using the DotPlot function.

Pearson correlations of FAP, PRSS3, GREM1, LGALS1, and PDPN with all other genes were computed across CAF single cells using log-normalized RNA expression. *P*-values were Benjamini-Hochberg adjusted (FDR), and results were limited to protein-coding genes. FDR < 0.05 was considered significant. For visualization, the top 10 positively correlated genes were shown as lollipop plots using ggplot2.

Single-cell pathway activity scores were calculated using decoupleR (2.8.0) (18) and PROGENy (19) model-derived pathway weights for target genes. Human interaction weights and the top 500 most responsive genes, ranked by *p*-value, were incorporated into a multivariate linear model (run_mlm) for inference following the package’s vignette. The resulting scores were scaled and visualized as heatmaps using ComplexHeatmap.

The transcription factor regulome analysis for CAFs was conducted using pySCENIC (0.12.1) (20) via the command-line interface. The raw count expression matrix served as the input, including only genes with nonzero counts per cell and detected in at least 1 per 1000 cells across the dataset, while mitochondrial and ribosomal genes were excluded. The Seurat matrix was exported as a loom file using the build_loom function from the R package SCopeLoomR (0.13.0) (21). The loom matrix was then used for gene regulatory network (GRN) inference via pyscenic grn with default settings. Next, the genes available in the cisTarget human hg38 motif databases were used (source) to define the modules of TF regulons with pyscenic ctx adding the parameter –mask_dropouts to the default ones. Finally, the regulon module score for each cell was computed with pyscenic aucell using default parameters. The AUC values were extracted and assigned to the Seurat object, and differential gene expression analysis was performed with the AUC assay using the FindAllMarkers function with default parameters. Differentially expressed genes with log2FC > 0.5 and adjusted *p*-value < 0.00001 were included. The AUC scores were scaled row wise to retain a Z-score, and relevant genes were visualized using ComplexHeatmap.

ScRNA-seq data from 5 previously published prostate cancer studies were downloaded from the Gene Expression Omnibus (GEO) under the accession numbers GSE137829 (22), GSE141445 (23), GSE176031 (24), GSE185344 (25), and GSE181294 (26). Pre-processing of metadata and quality control was performed on each dataset individually, and cells with less than 600 UMI counts, 300 gene counts, less than 10 total counts across each dataset, and cells with > 20% of counts originating from mitochondrial genes were excluded. Doublets were removed using DoubletFinder (2.0.4) (27) following the tutorial from <https://biostatsquid.com/doubletfinder-tutorial/>, and the batches were merged into a Seurat object. The Seurat object was preprocessed as described above, and the batches were integrated using Harmony integration. ElbowPlot was used to define the number of dimensions for RunUMAP, and FindNeighbors was conducted using the harmony reduction. The clustering was done with the FindClusters function using the Louvain algorithm, and clusters with more than 80% of cells originating from a single sample were discarded. This resulted in the exclusion of 2 clusters (3057 cells). To annotate the data, gene markers for 8 cell types were gathered from the original publications of the integrated datasets. Cell types were annotated using the scType (28) tutorial, and epithelial and fibroblast cells were extracted for further analysis. The fibroblasts/epithelial cells were integrated with the CAFs, preprocessed as above, and visualized in a UMAP. The unscaled average expression of selected markers was visualized using the DotPlot function, and the kernel density estimates were visualized using Nebulosa.

**References**

1. Bankhead P, Loughrey MB, Fernández JA, Dombrowski Y, McArt DG, Dunne PD, et al. QuPath: Open source software for digital pathology image analysis. *Scientific Reports 2017 7:1*. 2017;7(1): 1–7. https://doi.org/10.1038/s41598-017-17204-5.

2. Hadley Wickham. ggplot2: Elegant Graphics for Data Analysis. *Journal of the Royal Statistical Society Series A: Statistics in Society*. 216AD;174(1): 245–246.

3. Murumägi A, Ungureanu D, Khan S, Arjama M, Välimäki K, Ianevski A, et al. Drug response profiles in patient-derived cancer cells across histological subtypes of ovarian cancer: real-time therapy tailoring for a patient with low-grade serous carcinoma. *British journal of cancer*. 2023;128(4): 678–690. https://doi.org/10.1038/S41416-022-02067-Z.

4. Yadav B, Pemovska T, Szwajda A, Kulesskiy E, Kontro M, Karjalainen R, et al. Quantitative scoring of differential drug sensitivity for individually optimized anticancer therapies. *Scientific reports*. 2014;4. https://doi.org/10.1038/SREP05193.

5. Potdar S, Ianevski F, Ianevski A, Tanoli Z, Wennerberg K, Seashore-Ludlow B, et al. Breeze 2.0: An interactive web-tool for visual analysis and comparison of drug response data. *Nucleic Acids Research*. 2023;51(1 W): W57–W61. https://doi.org/10.1093/NAR/GKAD390.

6. Mpindi JP, Yadav B, Östling P, Gautam P, Malani D, Murumägi A, et al. Consistency in drug response profiling. *Nature*. 2016;540(7631): E5–E6. https://doi.org/10.1038/NATURE20171.

7. Dini A, Barker H, Piki E, Sharma S, Raivola J, Murumägi A, et al. A multiplex single-cell RNA-Seq pharmacotranscriptomics pipeline for drug discovery. *Nature Chemical Biology 2024 21:3*. 2024;21(3): 432–442. https://doi.org/10.1038/s41589-024-01761-8.

8. Frankish A, Diekhans M, Ferreira AM, Johnson R, Jungreis I, Loveland J, et al. GENCODE reference annotation for the human and mouse genomes. *Nucleic acids research*. 2019;47(D1): D766–D773. https://doi.org/10.1093/NAR/GKY955.

9. R Core Team. *R: a language and environment for statistical computing*. 2023. https://www.R-project.org

10. Hao Y, Stuart T, Kowalski MH, Choudhary S, Hoffman P, Hartman A, et al. Dictionary learning for integrative, multimodal and scalable single-cell analysis. *Nature Biotechnology 2023 42:2*. 2023;42(2): 293–304. https://doi.org/10.1038/s41587-023-01767-y.

11. Tirosh I, Izar B, Prakadan SM, Wadsworth MH, Treacy D, Trombetta JJ, et al. Dissecting the multicellular ecosystem of metastatic melanoma by single-cell RNA-seq. *Science (New York, N.Y.)*. 2016;352(6282): 189–196. https://doi.org/10.1126/SCIENCE.AAD0501.

12. Korsunsky I, Millard N, Fan J, Slowikowski K, Zhang F, Wei K, et al. Fast, sensitive and accurate integration of single-cell data with Harmony. *Nature Methods 2019 16:12*. 2019;16(12): 1289–1296. https://doi.org/10.1038/s41592-019-0619-0.

13. Traag VA, Waltman L, van Eck NJ. From Louvain to Leiden: guaranteeing well-connected communities. *Scientific Reports*. 2019;9(1): 1–12. https://doi.org/10.1038/s41598-019-41695-z.

14. Gu Z. Complex heatmap visualization. *iMeta*. 2022;1(3): e43. https://doi.org/10.1002/IMT2.43.

15. Peterson H, Kolberg L, Raudvere U, Kuzmin I, Vilo J. gprofiler2 -- an R package for gene list functional enrichment analysis and namespace conversion toolset g: Profiler. *F1000Research*. 2020;9. https://doi.org/10.12688/f1000research.24956.2.

16. Alquicira-Hernandez J, Powell JE. Nebulosa recovers single-cell gene expression signals by kernel density estimation. *Bioinformatics*. 2021;37(16): 2485–2487. https://doi.org/10.1093/BIOINFORMATICS/BTAB003.

17. Yang D, Liu J, Qian H, Zhuang Q. Cancer-associated fibroblasts: from basic science to anticancer therapy. *Experimental & Molecular Medicine*. 2023;55(7): 1322. https://doi.org/10.1038/S12276-023-01013-0.

18. Badia-I-Mompel P, Vélez Santiago J, Braunger J, Geiss C, Dimitrov D, Müller-Dott S, et al. decoupleR: ensemble of computational methods to infer biological activities from omics data. *Bioinformatics Advances*. 2022;2(1). https://doi.org/10.1093/BIOADV/VBAC016.

19. Schubert M, Klinger B, Klünemann M, Sieber A, Uhlitz F, Sauer S, et al. Perturbation-response genes reveal signaling footprints in cancer gene expression. *Nature Communications 2017 9:1*. 2018;9(1): 1–11. https://doi.org/10.1038/s41467-017-02391-6.

20. Aibar S, González-Blas CB, Moerman T, Huynh-Thu VA, Imrichova H, Hulselmans G, et al. SCENIC: single-cell regulatory network inference and clustering. *Nature Methods 2017 14:11*. 2017;14(11): 1083–1086. https://doi.org/10.1038/nmeth.4463.

21. Aerts Lab. *ScopeLoomR*. GitHub. GitHub; https://github.com/aertslab/SCopeLoomR/ [Accessed 14th January 2025].

22. Dong B, Miao J, Wang Y, Luo W, Ji Z, Lai H, et al. Single-cell analysis supports a luminal-neuroendocrine transdifferentiation in human prostate cancer. *Communications biology*. 2020;3(1). https://doi.org/10.1038/S42003-020-01476-1.

23. Chen S, Zhu G, Yang Y, Wang F, Xiao YT, Zhang N, et al. Single-cell analysis reveals transcriptomic remodellings in distinct cell types that contribute to human prostate cancer progression. *Nature cell biology*. 2021;23(1): 87–98. https://doi.org/10.1038/S41556-020-00613-6.

24. Song H, Weinstein HNW, Allegakoen P, Wadsworth MH, Xie J, Yang H, et al. Single-cell analysis of human primary prostate cancer reveals the heterogeneity of tumor-associated epithelial cell states. *Nature communications*. 2022;13(1). https://doi.org/10.1038/S41467-021-27322-4.

25. Wong HY, Sheng Q, Hesterberg AB, Croessmann S, Rios BL, Giri K, et al. Single cell analysis of cribriform prostate cancer reveals cell intrinsic and tumor microenvironmental pathways of aggressive disease. *Nature Communications*. 2022;13(1): 6036. https://doi.org/10.1038/S41467-022-33780-1.

26. Hirz T, Mei S, Sarkar H, Kfoury Y, Wu S, Verhoeven BM, et al. Dissecting the immune suppressive human prostate tumor microenvironment via integrated single-cell and spatial transcriptomic analyses. *Nature Communications 2023 14:1*. 2023;14(1): 1–20. https://doi.org/10.1038/s41467-023-36325-2.

27. McGinnis CS, Murrow LM, Gartner ZJ. DoubletFinder: Doublet Detection in Single-Cell RNA Sequencing Data Using Artificial Nearest Neighbors. *Cell Systems*. 2019;8(4): 329-337.e4. https://doi.org/10.1016/J.CELS.2019.03.003.

28. Ianevski A, Giri AK, Aittokallio T. Fully-automated and ultra-fast cell-type identification using specific marker combinations from single-cell transcriptomic data. *Nature Communications 2022 13:1*. 2022;13(1): 1–10. https://doi.org/10.1038/s41467-022-28803-w.
